# Soluble CD163 Is a Predictor of Mortality in Patients With Decompensated Cirrhosis

**DOI:** 10.3389/fmed.2021.698502

**Published:** 2021-07-15

**Authors:** Yue Zhang, Chenkai Huang, Yuan Nie, Qi Liu, Nanxi Xiao, Linxiang Liu, Xuan Zhu

**Affiliations:** Department of Gastroenterology, The First Affiliated Hospital of Nanchang University, Nanchang, China

**Keywords:** decompensated cirrhosis, soluble CD163, prognosis, CTP score, MELD score, ALBI score

## Abstract

**Background:** Soluble CD163 (sCD163) is a scavenger receptor membrane protein expressed almost exclusively on Kupffer cells and other macrophages. It was found to be associated with the severity of liver cirrhosis. The aim of the present study was to determine whether the novel biomarker sCD163 predicts outcomes in patients with decompensated cirrhosis.

**Materials and Methods:** A single-center, observational, prospective study with 345 decompensated cirrhosis patients was conducted in the Gastroenterology Department between January 2017 and December 2020. Their plasma samples were tested by enzyme-linked immunosorbent assay (ELISA) for sCD163 within 24 hours of admission. These patients were followed up at 28 days, 3 months and 6 months. The independent risk factors were identified with uni- and multivariate logistic regression analyses. We evaluated the predictive performance of the new scoring system (including sCD163) and the original scoring system.

**Results:** The sCD163 level was significantly higher in non-surviving patients than in surviving patients. Positive associations were found between sCD163 levels and the Child-Turcotte-Pugh (CTP), Model for End-Stage Liver Disease (MELD) and albumin-bilirubin (ALBI) scores. Logistic regression confirmed that sCD163 was an independent risk factor for 28-day, 3-month, and 6-month mortality. The areas under the receiver operating characteristic curves (AUROCs) of the use of sCD163 for the prediction of 28-day, 3-month, and 6-month mortality were relatively higher (AUROCs: 0.856; 0.823 and 0.811, respectively). The AUROCs of the new scores obtained by adding sCD163 to the original scoring systems (CTP + sCD163, MELD + sCD163 and ALBI + sCD163) showed that the new scoring systems had better predictive performance than the original scoring systems at all time points (*P* < 0.001).

**Conclusion:** sCD163 is a prognostic predictor of short-term and long-term outcomes in decompensated cirrhosis patients. Accordingly, the addition of sCD163 to the original clinical scoring systems improved their prognostic performance.

## Introduction

Liver cirrhosis is the end stage of various liver diseases, such as alcoholic steatohepatitis, chronic viral hepatitis, autoimmune hepatitis, non-alcoholic fatty liver disease and some diseases with genetic etiologies. Liver cirrhosis causes more than one million deaths per year, and the prevalence is increasing (1). The etiology of liver cirrhosis varies among regions, and infection with hepatitis B virus (HBV) is the main cause of liver cirrhosis in China (2). A number of complications can occur in patients with cirrhosis, including upper gastrointestinal bleeding, the rapid accumulation of ascites, hepatic encephalopathy (HE) and bacterial infections, which are collectively known as acute decompensation (AD) (3). The occurrence of decompensated cirrhosis (DeCi) is associated with increased mortality (4). Cirrhosis imposes a substantial health burden in many countries. Although effective interventions for the prevention and treatment of hepatitis B and C are available, they are still the main causes of cirrhosis worldwide, particularly in low-income countries (5). Various clinical scores have been established as predictors of mortality in DeCi patients, such as the Child-Turcotte-Pugh (CTP) score, the Model for End-stage Liver Disease (MELD) score and albumin-bilirubin (ALBI) score. The CTP score was widely used as a liver-specific score nearly 50 years ago and was calculated based on the albumin level, prothrombin time, serum bilirubin level, ascites and HE (6). The MELD score is determined based on the creatinine level, bilirubin level and international normalized ratio (INR), and it was first used to evaluate outcomes in patients undergoing transjugular intrahepatic portosystemic shunt (TIPS) placement (7). The ALBI score is calculated based on the levels of bilirubin and albumin and has been used to assess liver function in patients with hepatocellular carcinoma (8). These prognostic scores are pragmatically based on widely available biochemical and clinical data; however, biomarkers of the mechanisms suspected of underlying the development of DeCi may shed light on the pathogenesis of this condition, improve the predictive ability of these scores, and suggest strategies for future rational treatments of DeCi.

CD163 is predominantly expressed on Kupffer cells, which are the resident macrophages in the liver and play a central role in the development of cirrhosis by modulating pro- and anti-inflammatory signals (9). Plasma soluble CD163 (sCD163) is regarded as a marker of circulating macrophage activation and is commonly elevated in patients with inflammatory diseases, including chronic liver diseases (10). A previous study confirmed that sCD163 is a prognostic parameter for overall survival in patients with hepatocellular carcinoma (HCC) and liver cirrhosis (9, 11). It has also been shown that sCD163 correlates with portal hypertension and liver dysfunction in cirrhosis patients (12, 13).

As the sCD163 serum level is an indicator of overall survival in cirrhosis patients, we postulated that it might have excellent predictive ability for their prognosis. The aim of our study was to examine the prognostic value of sCD163 and compare it to that of established models in a prospective cohort of patients with cirrhosis.

## Materials and Methods

### Study Patients

Patients diagnosed with DeCi at the First Affiliated Hospital of Nanchang University between January 2017 and December 2020 were included. The inclusion criteria were age ≥18 years and a diagnosis of DeCi. The exclusion criteria were hepatocellular carcinoma, compensated cirrhosis, infection with human immunodeficiency virus and other severe chronic extrahepatic diseases. This prospective study was approved by the ethics committee of the First Affiliated Hospital of Nanchang University (No. IIT [2017] 009). The study was performed in accordance with the principles of the Declaration of Helsinki.

### Definitions

In all patients, the diagnosis of DeCi had already been made based on biochemical results, radiologic evidence or biopsy before inclusion, including severe complications of cirrhosis, such as hepatorenal syndrome, massive ascites, gastrointestinal bleeding, HE or spontaneous bacterial peritonitis (SBP). The criteria were defined based on the evidence-based clinical practice guidelines for liver cirrhosis published in 2015 (14). The CTP and MELD scores were evaluated based on laboratory parameters, clinical examinations and the results of abdominal ultrasound, CT or MRI examinations (6, 7). ALBI scores were calculated based on the levels of albumin and bilirubin with the following formula: 0.66 × log(TBiL [μmol/L])−0.085(albumin [g/L]) (8). Acute decompensation was defined as acute development of one or more major complications of liver disease, including acute hepatic encephalopathy, acute development of large ascites (within <2 weeks; ultrasound ascites volume ≧150 mL), acute gastrointestinal hemorrhage, and bacterial infection (15). Acute on chronic liver failure (ACLF) was defined according to the European Association for the Study of the Liver-Chronic Liver Failure (EASL-CLIF) criteria (15).

### Measurement of Plasma sCD163

Soluble CD163 was collected on the day of inclusion in the study. Blood samples were obtained and stored at −80 °C until biochemical analysis. Soluble CD163 is resistant to repeated freezing and thawing (16). Soluble CD163 levels were measured by enzyme-linked immunosorbent assay (ELISA) (R&D Systems) according to the manufacturer's protocol. We previously established a reference interval (0.69–3.86 mg/L) for sCD163 in a large cohort of healthy individuals with the same assay (17). The measurement of sCD163 was performed by professional staff at the Department of Clinical Biochemistry.

### Data Collection

Data for the included patients, such as demographics, biochemical results, blood hematological index values, the etiology of liver cirrhosis and the degree of ascites, were all extracted from the medical records of the Department of Gastroenterology. The sCD163 results were collected from a datasheet generated by laboratory professional staff. The study endpoints were 28-day, 3-month and 6-month mortality. For discharged patients, prognostic information was confirmed in the medical records, via phone contact or during visits.

### Statistical Analysis

All statistical analyses were performed with SPSS software version 20.0 (SPSS Inc., Chicago, IL). Discrete variables are presented as counts (percentage), and continuous variables are presented as the means (SDs). Non-normally distributed variables were summarized as the medians (interquartile ranges; IQRs). The chi-squared test was used for categorical variables, Student's *t*-test was used for normally distributed continuous variables, and the Mann-Whitney U test was used for continuous variables that were not normally distributed. The correlations between the sCD163 level and the scores were assessed with the Spearman rank correlation test. A confirmatory analysis was performed by estimating the area under the receiver operating characteristic (ROC) curve (AUROC). The Delong test was used to compare the AUROCs. *P* values < 0.05 were considered statistically significant.

## Results

### Characteristics of the Included Patients

**Figure 1:**
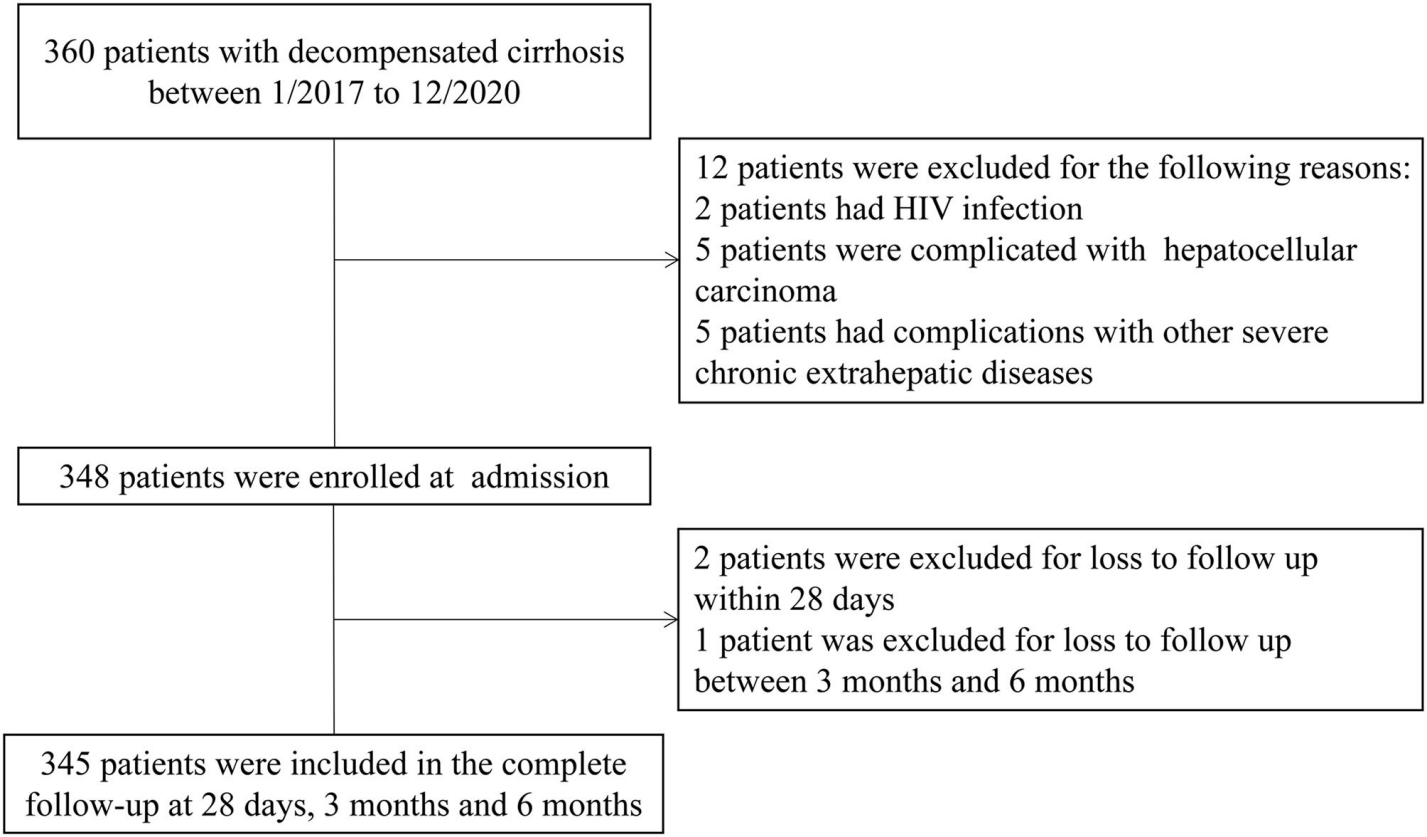
The flowchart in the study.

Between January 2017 and December 2020, 345 patients with liver cirrhosis were included in the present study and the flowchart is shown in Figure 1. The patient characteristics are presented in Table 1. The mean (±SD) age of the 345 patients was 55.22 (±11.86) years. There was a male predominance (73.3%). HBV infection was the most common etiology in these cirrhosis patients. The main reason for hospitalization among the DeCi patients was variceal bleeding (86.9%). ACLF patients accounted for 15.7% of the patients with cirrhosis, and the ACLF grade distribution was as follows: grade 1 (7.5%), grade 2 (5.5%), and grade 3 (2.6%). The most common degree of ascites was mild (26.7%), followed by moderate (15.4%) and severe (14.5%). In these patients with cirrhosis, a total of 22 (6.4%) patients received mechanical ventilation. In total, 56 (16.2%), 84 (24.3%), and 99 (28.7%) patients died within 28 days, 3 months, and 6 months, respectively. The causes of death at 6 months were as follows: 45 (45.5%) patients had hemorrhagic shock, 17 (17.2%) patients had HE, 15 (15.2%) patients had respiratory failure, 10 (10.1%) patients had pulmonary infections, 7 (7.0%) patients had cardiogenic shock, 1 (1.0%) patient had a ruptured thoracic aortic aneurysm, 1 (1.0%) patient had acute myocardial infarction, and 3 (3.0%) patients had an uncertain cause of death. The causes of death at 6 months are summarized in Supplementary Table 1.

**Table 1:** Characteristics of the included patients.

	**Patients with decompensated cirrhosis (*n* = 345)**
Age, mean ±SD	55.22 ± 11.86
Gender, *n* (%)	
Male	253 (73.3)
Female	92 (26.7)
Liver cirrhosis etiology, *n* (%)	
Hepatitis B Virus	219 (63.5)
Alcoholic liver disease	55 (15.9)
Hepatitis C Virus	13 (3.8)
Primary biliary cirrhosis	7 (2.0)
Schistosomiasis cirrhosis	18 (5.2)
Others	33 (9.6)
The reason for hospitalization, *n* (%)	
Variceal bleeding	300 (86.9)
Hepatic encephalopathy	19 (5.5)
Infection	19 (5.5)
Ascites	5 (1.4)
Others	2 (0.7)
ACLF patients, *n* (%)	54 (15.6)
ACLF grade 1	26 (7.5)
ACLF grade 2	19 (5.5)
ACLF grade 3	9 (2.6)
Ascites degree, *n* (%)	
Mild	92 (26.7)
Moderate	53 (15.4)
Severe	50 (14.5)
Mechanical ventilation, *n* (%)	22 (6.4)

*ACLF, Acute-on-chronic liver failure; SD, Standard Deviation*.

### Laboratory Characteristics in the Non-surviving and Surviving Groups

The laboratory characteristics of the patients are presented in Table 2. DeCi patients were grouped into non-surviving and surviving groups based on their 28-day, 3-month, and 6-month outcomes. The INR, creatinine level, bilirubin level, WBC count, prothrombin time (PT), aspartate transaminase (AST) level, gamma-glutamyl transferase (GGT) level, alkaline phosphatase (ALP) level, CTP score, MELD score, ALBI score and sCD163 level were significantly higher in the non-surviving group than in the surviving group at all time points (all *P* < 0.05), whereas the PO2/FiO2 and albumin were significantly lower in the non-surviving group than in the surviving group (all *P* < 0.05). The ALT level was significantly higher in the non-surviving group than in the surviving group at 3 months and 6 months (*P* < 0.05), but the difference was not significant at 28 days (*P* > 0.05). No significant difference was detected in the platelet count, mean arterial pressure (MAP) or serum sodium level (all *P* > 0.05).

**Table 2:** Clinical characteristics and prognostic model between non-surviving and surviving patients.

**Variables**	**28-days**		***P***	**3-months**		***P***	**6-months**		***P***
	**Non-Survivors** **(*n* = 56)**	**Survivors** **(*n* = 289)**		**Non-Survivors** **(*n* = 84)**	**Survivors** **(*n* = 261)**		**Non-Survivors** **(*n* = 99)**	**Survivors** **(*n* = 246)**	
INR	1.46 (1.24–1.87)	1.31 (1.19–1.49)	**<0.001**	1.43 (1.27–1.75)	1.30 (1.18–1.48)	**<0.001**	1.38 (1.24–1.71)	1.31 (1.19–1.48)	**0.001**
Creatinine, umol/L	97.80 (72.15–139.60)	72.40 (59.60–90.45)	**<0.001**	93.75 (64.05–138.78)	72.00 (59.60–85.55)	**<0.001**	89.20 (62.10–134.60)	71.90 (59.60–85.48)	**<0.001**
Bilirubin, umol/L	30.85 (19.13–78.23)	22.80 (14.90–38.00)	**0.005**	26.75 (17.05–60.95)	22.70 (14.55–37.60)	**0.006**	26.60 (17.40–58.60)	22.15 (14.28–37.78)	**0.004**
Platelet, 10*9/L	73.00 (31.50–113.50)	62.00 (40.00–92.50)	0.567	70.00 (33.50–104.75)	61.00 (40.50–91.50)	0.532	70.00 (35.00–108.00)	60.50 (40.00–90.25)	0.0.369
WBC, 10*9/L	8.94 (5.29–15.08)	6.21 (3.86–8.96)	**<0.001**	8.35 (5.08–13.95)	6.12 (3.84–8.77)	**<0.001**	6.97 (4.71–13.45)	6.22 (3.82–8.94)	**0.004**
PT	15.70 (13.90–21.68)	14.80 (13.15–16.45)	**0.002**	15.65 (13.93–20.05)	14.70 (13.10–16.35)	**<0.001**	15.20 (13.90–19.90)	14.75 (13.08–16.40)	**0.002**
MAP, mmHg	82.33 (76.33–89.00)	83.00 (79.00–89.00)	0.248	82.33 (77.33–88.92)	83.00 (79.00–89.00)	0.342	82.67 (77.33–89.00)	82.83 (78.92–89.00)	0.627
PO2/FiO2	367.79 ± 134.70	410.22 ± 117.19	**0.016**	380.40 ± 133.42	410.72 ± 116.05	**0.046**	378.58 ± 134.33	413.30 ± 114.01	**0.016**
ALT, IU/L	27.50 (17.25–73.75)	25.00 (17.00–40.00)	0.087	29.50 (18.00–72.50)	24.00 (17.00–38.00)	**0.025**	28.00 (17.00–71.00)	24.00 (17.00–38.00)	**0.020**
AST, IU/L	78.00 (39.00–189.25)	38.00 (26.00–57.00)	**<0.001**	69.00 (38.25–189.25)	37.00 (26.00–53.50)	**<0.001**	60.00 (37.00–169.00)	36.50 (26.00–53.00)	**<0.001**
GGT, IU/L	42.00 (18.75–110.75)	23.00 (14.00–50.00)	**0.002**	39.00 (21.00–104.25)	22.00 (14.00–48.00)	**<0.001**	37.00 (17.00–96.00)	22.00 (14.00–48.00)	**0.001**
ALP, IU/L	102.00 (64.75–167.00)	69.00 (53.00–96.00)	**<0.001**	95.50 (63.25–164.00)	68.00 (53.00–93.50)	**<0.001**	92.00 (64.00–164.00)	67.00 (53.00–93.25)	**<0.001**
Albumin, g/L	25.86 ± 4.59	28.93 ± 4.95	**<0.001**	26.07 ± 4.94	29.19 ± 4.81	**<0.001**	26.41 ± 5.03	29.25 ± 4.78	**<0.001**
Serum sodium	139.00 (134.00–143.18)	138.10 (136.00–141.00)	0.385	138.00 (134.00–141.90)	138.40 (136.00–143.00)	0.700	138.0 (134.00–141.00)	138.35 (136.00–141.00)	0.501
CTP score	10.00 (8.00–11.00)	8.00 (7.00–9.00)	**<0.001**	9.00 (8.00–10.00)	8.00 (7.00–9.00)	**<0.001**	9.00 (8.00–10.00)	8.00 (7.00–9.00)	**<0.001**
MELD score	14.50 (11.00–23.00)	11.00 (9.00–14.00)	**<0.001**	14.00 (11.00–20.00)	10.00 (9.00–14.00)	**<0.001**	14.00 (10.00–19.00)	10.00 (9.00–14.00)	**<0.001**
ALBI score	−1.14 ± 0.58	−1.54 ± 0.48	**<0.001**	−1.19 ± 0.56	−1.57 ± 0.47	**<0.001**	−1.27 ± 0.55	−1.58 ± 0.48	**<0.001**
sCD163, mg/L	11.35 (7.10–20.35)	4.16 (3.12–5.94)	**<0.001**	8.55 (6.01–14.57)	4.08 (3.12–5.48)	**<0.001**	8.35 (5.23–13.87)	3.84 (3.06–5.28)	**<0.001**

*P value < 0.05 was considered significant and was indicated in bold*.

*INR, International normalized ratio; WBC, White blood cell count; PT, Prothrombin time; MAP, Mean arterial pressure; ALT, Alanine aminotransferase; AST, Aspartate aminotransferase; GGT, Gamma-glutamyl transpeptidase; ALP, Alkaline phosphatase; CTP, Child-Turcotte-Pugh; MELD, model for end-stage liver disease; ALBI, Albumin-Bilirubin; sCD163, soluble CD163*.

### sCD163 Level Is Correlated With the CTP, MELD and ALBI Scores

The sCD163 level was confirmed to be significantly positively correlated with the CTP, MELD and ALBI scores in all patients (rho = 0.389; rho = 0.324; rho = 0.385, respectively; all *P* < 0.001). As expected, we observed that the sCD163 level was correlated with the clinical scores in the patients who survived and did not survive at all time points, as shown in Table 3.

**Table 3:** Correlations between sCD163 and scores for liver disease severity (CTP, MELD and ALBI scores).

**Correlations** **(Spearman rho(*****p*****-value))**		**All patients (*n* = 345)**	**28-day**		**3-month**		**6-month**	
			**Surviving**	**Non-surviving**	**Surviving**	**Non-surviving**	**Surviving**	**Non-surviving**
			**(*n* = 289)**	**(*n* = 56)**	**(*n* = 261)**	**(*n* = 84)**	**(*n* = 246)**	**(*n* = 99)**
sCD163	CTP	0.389 (< **0.001**)	0.319 (< **0.001**)	0.366 (< **0.001**)	0.320 (< **0.001**)	0.352 (< **0.001**)	0.301 (< **0.001**)	0.359 (< **0.001**)
	MELD	0.324 (< **0.001**)	0.272 (< **0.001**)	0.372 (< **0.001**)	0.243 (< **0.001**)	0.323 (< **0.001**)	0.242 (< **0.001**)	0.315 (< **0.001**)
	ALBI	0.385 (< **0.001**)	0.354 (< **0.001)**	0.376 (< **0.001**)	0.345 (< **0.001**)	0.352 (< **0.001**)	0.316 (< **0.001**)	0.381 (< **0.001**)

*P value < 0.05 was considered significant and was indicated in bold*.

*CTP, Child-Turcotte-Pugh; MELD, model for end-stage liver disease; ALBI, Albumin-Bilirubin; sCD163, soluble CD163*.

### Predictive Factors Related to the Prognosis of Patients With Decompensated Cirrhosis

As shown in Table 4, the univariate logistic regression analysis of 28-day mortality showed that age >60 years, the presence of ACLF, INR, creatinine level, bilirubin level, WBC count, PT, PO2/FiO2, ALT level, AST level, ALP level and sCD163 level were relevant risk factors (all *P* < 0.05), and albumin was a protective factor (*P* < 0.05). Multivariate logistic regression analysis identified that age >60 years and the levels of bilirubin, ALP and sCD163 were independent risk factors for 28-day mortality (OR: 2.291, 95% CI: 1.054–4.981; OR: 1.007, 95% CI: 1.001–1.013; OR: 1.006, 95% CI: 1.002–1.010; OR: 1.297, 95% CI: 1.187–1.416, respectively), and albumin was a protective factor (OR: 0.893, 95% CI: 0.811–0.983). The multivariate logistic regression analysis of 3-month mortality showed that age >60 years and the levels of bilirubin, ALP and sCD163 were independent risk factors for 3-month mortality (OR: 1.930, 95% CI: 0.989–3.766; OR: 1.008, 95% CI: 1.001–1.015; OR: 1.005, 95% CI: 1.001–1.009; OR: 1.279, 95% CI: 1.174–1.394, respectively), and albumin was a protective factor (OR: 0.886, 95% CI: 0.817–0.962). The multivariate logistic regression analysis of 6-month mortality confirmed that the levels of bilirubin, ALP and sCD163 were independent risk factors for 6-month mortality (OR: 1.007, 95% CI: 1.000–1.014; OR: 1.006, 95% CI: 1.001–1.011; OR: 1.300, 95% CI: 1.187–1.423, respectively), and albumin was a protective factor (OR: 0.893, 95% CI: 0.827–0.963).

**Table 4:** Independent predictors for 28-day, 3-month and 6-month mortality in univariate and multivariate analysis.

	**28 days**			**3 months**			**6 months**		
	**Univariate**		**Multivariate**	**Univariate**		**Multivariate**	**Univariate**		**Multivariate**
	**OR (95% CI)**	***P*-value**	**OR (95% CI)**	**OR (95% CI)**	***P*-value**	**OR (95% CI)**	**OR (95% CI)**	***P*-value**	**OR (95% CI)**
Age									
≤ 60	Reference		Reference	Reference		Reference	Reference		
>60	1.994 (1.117–3.559)	**0.020**	2.291 (1.054–4.981)	1.836 (1.110–3.037)	**0.018**	1.930 (0.989–3.766)	1.718 (1.062–2.778)	**0.027**	
Sex									
Female	Reference			Reference			Reference		
Male	0.804 (0.429–1.506)	0.496		1.219 (0.689–2.157)	0.496		1.288 (0.748–2.217)	0.361	
The presence of ACLF									
No. ACLF	Reference			Reference			Reference		
ACLF	4.636 (2.417–8.892)	**<0.001**		4.956 (2.693–9.123)	**<0.001**		5.333 (2.892–9.836)	**<0.001**	
INR	4.859 (2.373–9.948)	**<0.001**		5.021 (2.492–10.117)	**<0.001**		3.905 (2.003–7.614)	**<0.001**	
Creatinine, umol/L	1.005 (1.001–1.009)	**0.021**		1.014 (1.008–1.020)	**<0.001**		1.013 (1.007–1.019)	**<0.001**	
Bilirubin, umol/L	1.009 (1.004–1.013)	**<0.001**	1.007 (1.001–1.013)	1.009 (1.004–1.014)	**<0.001**	1.008 (1.001–1.015)	1.008 (1.004–1.013)	**<0.001**	1.007 (1.000–1.014)
Platelet, 10*9/L	1.002 (0.998–1.006)	0.286		1.003 (1.000–1.006)	0.065		1.003 (1.000–1.007)	0.059	
WBC, 10*9/L	1.084 (1.040–1.131)	**<0.001**		1.071 (1.029–1.114)	**0.001**		1.058 (1.019–1.098)	**0.003**	
PT	1.149 (1.080–1.223)	**<0.001**		1.136 (1.070–1.206)	**<0.001**		1.116 (1.054–1.182)	**<0.001**	
MAP, mmHg	0.978 (0.950–1.007)	0.131		0.990 (0.966–1.015)	0.423		0.996 (0.973–1.020)	0.756	
PO_2_/FiO_2_	0.997 (0.995–0.999)	**0.017**		0.998 (0.996–1.000)	**0.047**		0.998 (0.996–1.000)	**0.017**	
ALT, IU/L	1.002 (1.000–1.004)	**0.017**		1.002 (1.000–1.004)	**0.035**		1.002 (1.001–1.004)	**0.009**	
AST, IU/L	1.002 (1.001–1.003)	**0.003**		1.002 (1.001–1.003)	**0.002**		1.002 (1.001–1.004)	**0.001**	
GGT, IU/L	1.002 (0.999–1.004)	0.173		1.002 (0.999–1.004)	0.172		1.003 (1.001–1.006)	**0.015**	
ALP, IU/L	1.006 (1.003–1.009)	**<0.001**	1.006 (1.002–1.010)	1.006 (1.003–1.010)	**<0.001**	1.005 (1.001–1.009)	1.007 (1.004–1.011)	**<0.001**	1.006 (1.001–1.011)
Albumin, g/L	0.875 (0.821–0.933)	**<0.001**	0.893 (0.811–0.983)	0.871 (0.823–0.922)	**<0.001**	0.886 (0.817–0.962)	0.883 (0.837–0.931)	**<0.001**	0.893 (0.827–0.963)
Serum sodium, mmol/L	1.023 (0.972–1.077)	0.380		1.008 (0.980–1.037)	0.576		1.006 (0.982–1.032)	0.611	
sCD163, mg/L	1.262 (1.179–1.351)	**<0.001**	1.297 (1.187–1.416)	1.281 (1.193–1.374)	**<0.001**	1.279 (1.174–1.394)	1.291 (1.200–1.387)	**<0.001**	1.300 (1.187–1.423)

*P value < 0.05 was considered significant and was indicated in bold*.

*INR, International normalized ratio; WBC, White blood cell count; PT, Prothrombin time; MAP, Mean arterial pressure; ALT, Alanine aminotransferase; AST, Aspartate aminotransferase; GGT, Gamma-glutamyl transpeptidase; ALP, Alkaline phosphatase; sCD163, soluble CD163*.

### Predictive Value of sCD163 in Decompensated Cirrhosis Patients

**Figure 2:**
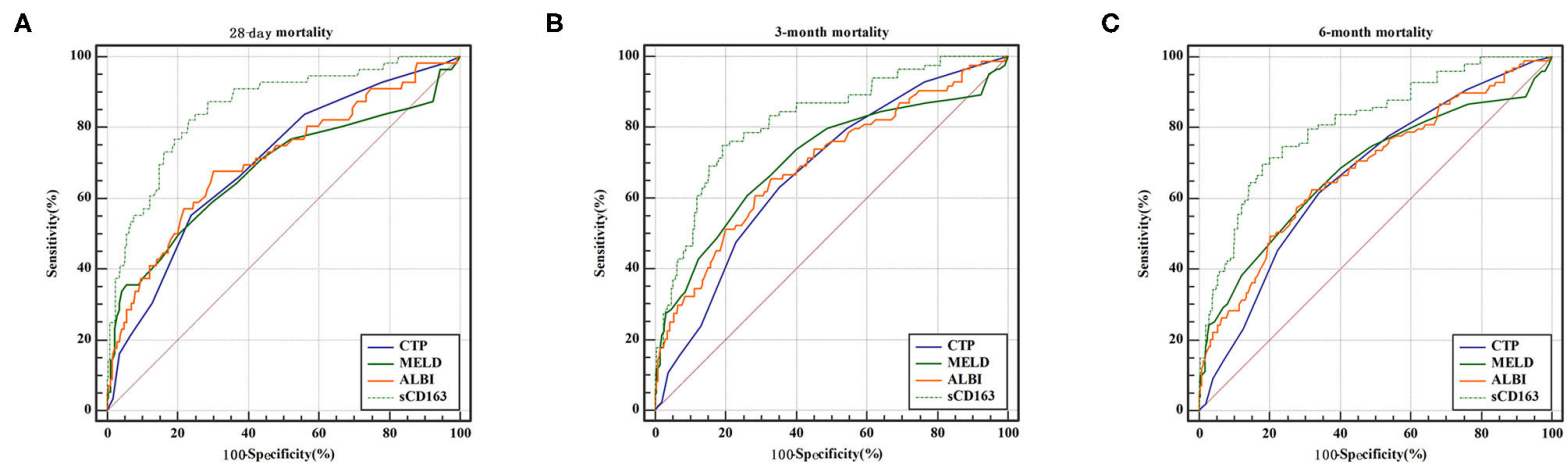
ROC curve analysis and comparison between sCD163 and prognostic scores for predicting 28-day, 3-month and 6-month mortality **(A)** ROC for 28-day; **(B)** ROC for 3-month; **(C)** ROC for 6-month.

We generated ROC curves to evaluate the effectiveness of the CTP score, MELD score, ALBI score, and sCD163 for the prediction of 28-day, 3-month and 6-month mortality. As shown in Table 5, the sCD163 level had the highest AUROCs at 28 days, 3 months and 6 months (AUROC = 0.856, 95% CI: 0.814–0.891; AUROC = 0.823, 95% CI: 0.779–0.862; and AUROC = 0.811, 95% CI: 0.766–0.851, respectively). The cutoff value for sCD163 for the prediction of 28-day mortality was 5.91, with a sensitivity of 83.93% and a specificity of 75.09%. The cutoff value for sCD163 for the prediction of 3-month mortality was 6.00, with a sensitivity of 75.00% and a specificity of 80.84%. The cutoff value for sCD163 for the prediction of 6-month mortality was 6.00, with a sensitivity of 69.70% and a specificity of 82.11%. The CTP score, MELD score, and ALBI score also had predictive value for mortality at 28 days, 3 months, and 6 months in DeCi patients (all *P* < 0.001). The ROC curves for sCD163 and the three prognostic scores are shown in Figure 2.

**Table 5:** The performance of sCD163 and the prognostic scores for predicting outcome at 28 days, 3 months and 6 months.

**Prognostic score**	**ROC area (95%CI)**	***P*-value**	**cut-off point**	**Sensitivity (%)**	**Specificity (%)**
**28-day mortality**					
CTP	0.699 (0.648–0.747)	**<0.001**	9.00	55.36	76.12
MELD	0.680 (0.628–0.729)	**<0.001**	18.00	35.71	94.46
ALBI	0.708 (0.657–0.755)	**<0.001**	−1.28	67.86	69.90
sCD163	0.856 (0.814–0.891)	**<0.001**	5.91	83.93	75.09
CTP + sCD163	0.861 (0.820–0.896)	**<0.001**	14.91	87.50	74.74
MELD + sCD163	0.827 (0.783–0.866)	**<0.001**	23.08	66.07	86.85
ALBI + sCD163	0.867 (0.827–0.901)	**<0.001**	4.40	87.50	75.09
**3-month mortality**					
CTP score	0.676 (0.623–0.725)	**<0.001**	8.00	63.10	64.75
MELD score	0.712 (0.661–0.759)	**<0.001**	13.00	60.71	73.95
ALBI score	0.708 (0.657–0.755)	**<0.001**	−1.28	67.86	69.90
sCD163	0.823 (0.779–0.862)	**<0.001**	6.00	75.00	80.84
CTP + sCD163	0.828 (0.784–0.886)	**<0.001**	14.91	76.19	77.78
MELD + sCD163	0.825 (0.781–0.864)	**<0.001**	18.32	80.95	73.56
ALBI + sCD163	0.836 (0.793–0.874)	**<0.001**	4.40	77.38	78.54
**6-month mortality**					
CTP score	0.666 (0.614–0.716)	**<0.001**	8.00	61.62	65.85
MELD score	0.681 (0.629–0.730)	**<0.001**	12.00	61.62	67.07
ALBI score	0.683 (0.631–0.731)	**<0.001**	−1.37	62.63	67.89
sCD163	0.811 (0.766–0.851)	**<0.001**	6.00	69.70	82.11
CTP + sCD163	0.810 (0.765–0.850)	**<0.001**	14.88	73.74	79.67
MELD+ sCD163	0.799 (0.753–0.840)	**<0.001**	18.32	75.76	74.80
ALBI + sCD163	0.821 (0.776–0.860)	**<0.001**	4.40	72.73	80.08

*P value < 0.05 was considered significant and was indicated in bold*.

*CTP, Child-Turcotte-Pugh; MELD, model for end-stage liver disease; ALBI, Albumin-Bilirubin; sCD163, soluble CD163*.

### Predictive Value Is Improved by Adding sCD163 to the Scores

**Figure 3:**
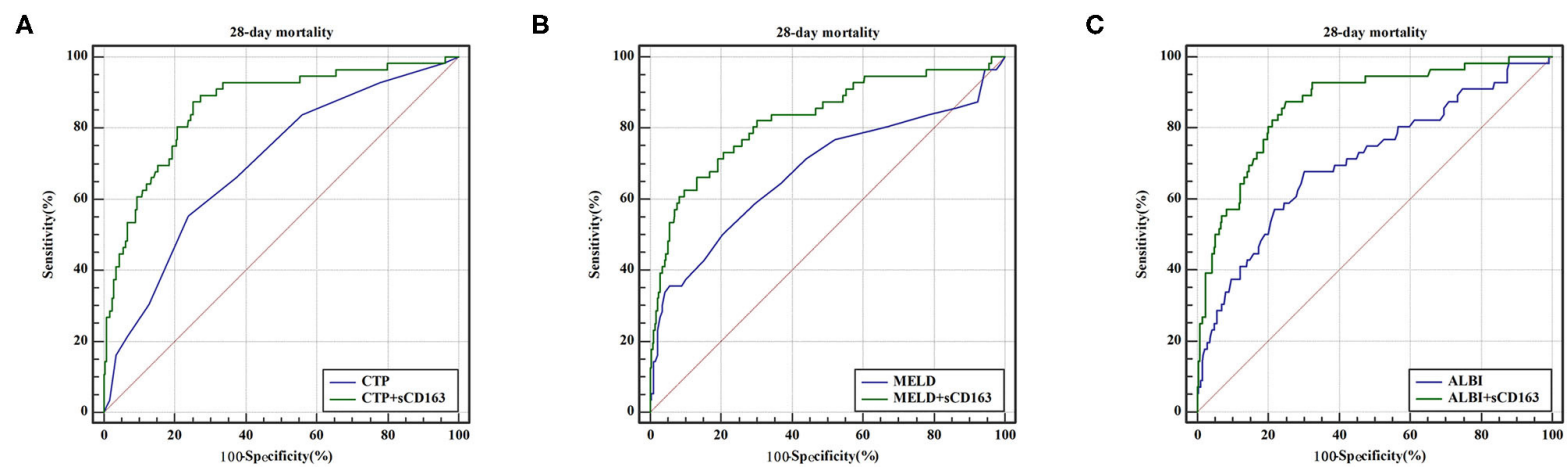
ROC curve analysis and comparison between new scores and original scores for predicting 28-day mortality **(A)** ROC for CTP and CTP+sCD163; **(B)** ROC for MELD and MELD+sCD163; **(C)** ROC for ALBI and ALBI+sCD163.

**Figure 4:**
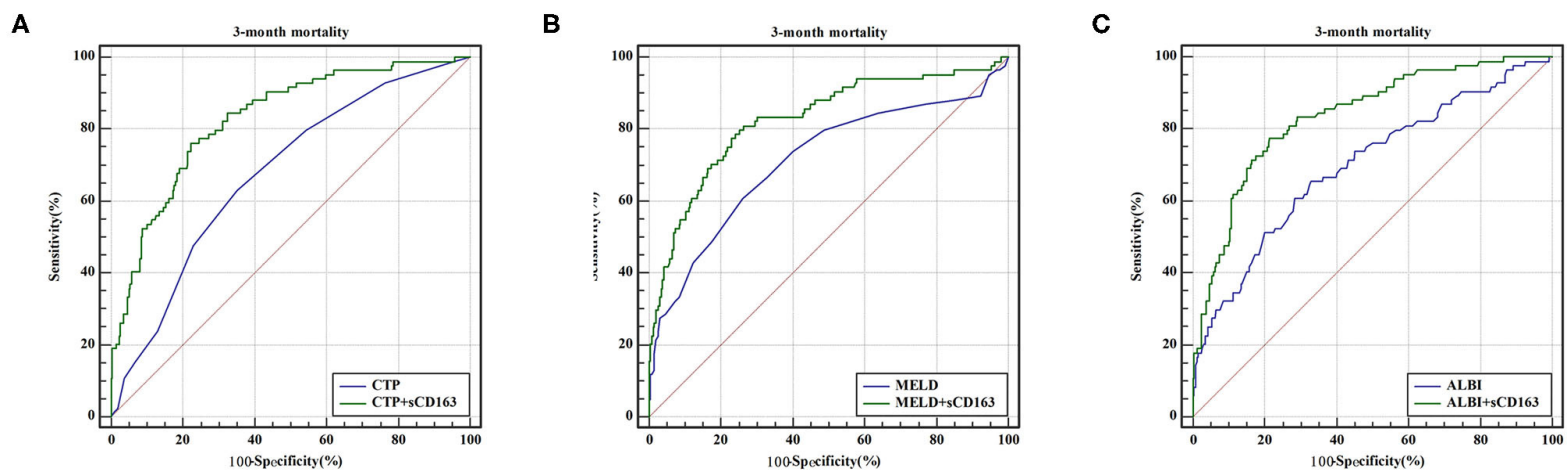
ROC curve analysis and comparison between new scores and original scores for predicting 3-month mortality **(A)** ROC for CTP and CTP+sCD163; **(B)** ROC for MELD and MELD+sCD163; **(C)** ROC for ALBI and ALBI+sCD163.

**Figure 5:**
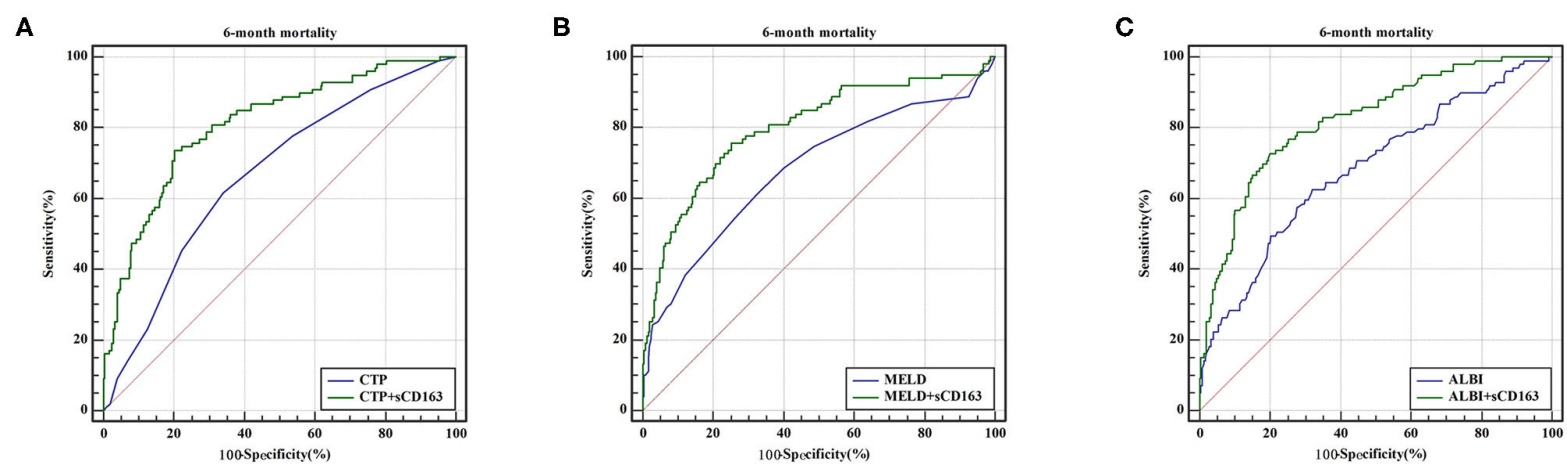
ROC curve analysis and comparison between new scores and original scores for predicting 6-month mortality **(A)** ROC for CTP and CTP+sCD163; **(B)** ROC for MELD and MELD+sCD163; **(C)** ROC for ALBI and ALBI+sCD163.

As indicated above, sCD163 had the best predictive value, and we established new scores (CTP + sCD163, MELD + sCD163 and ALBI + sCD163) to improve the predictive value of the original scores with the addition of sCD163. The AUROCs of the new scores exhibited excellent predictive value at 28 days, 3 months and 6 months (all *P* < 0.001), and the analysis is shown in Table 5. At 28 days, the AUROCs of the CTP + sCD163 score, MELD + sCD163 score and ALBI + sCD163 score were higher than those of the CTP, MELD and ALBI scores (difference between areas = 0.162, 95% CI = 0.097–0.227, *P* < 0.001; difference between areas = 0.147, 95% CI = 0.085–0.209, *P* < 0.001; difference between areas = 0.159, 95% CI = 0.072–0.247, *P* < 0.001, respectively). At 3 months, the AUROCs of the CTP + sCD163 score, MELD + sCD163 score and ALBI + sCD163 score were significantly greater than those of the CTP, MELD and ALBI scores (difference between areas = 0.152, 95% CI = 0.098–0.206, *P* < 0.001; difference between areas = 0.114, 95% CI = 0.067–0.161, *P* < 0.001; difference between areas = 0.139, 95% CI = 0.068–0.210, *P* < 0.001, respectively). At 6 months, the AUROCs of the CTP + sCD163 score, MELD + sCD163 score and ALBI + sCD163 score were greater than those of the CTP, MELD and ALBI scores (difference between areas = 0.144, 95% CI = 0.095–0.192, *P* < 0.001; difference between areas = 0.118, 95% CI = 0.075–0.161, *P* < 0.001; difference between areas = 0.138, 95% CI = 0.075–0.202, *P* < 0.001, respectively). The AUROC analysis is summarized in Table 6. The ROC curves are shown in Figure 3 (28 days), Figure 4 (3 months) and Figure 5 (6 months).

**Table 6:** The predictive value of various scores with adding sCD163.

**Prognostic score**	**Difference between areas (95%CI)**	***P*-value**
**28-day mortality**		
CTP + sCD163 vs. CTP	0.162 (0.097–0.227)	**<0.001**
MELD + sCD163 vs. MELD	0.147 (0.085–0.209)	**<0.001**
ALBI + sCD163 vs. ALBI	0.159 (0.072–0.247)	**<0.001**
**3-month mortality**		
CTP + sCD163 vs. CTP	0.152 (0.098–0.206)	**<0.001**
MELD + sCD163 vs. MELD	0.114 (0.067–0.161)	**<0.001**
ALBI + sCD163 vs. ALBI	0.139 (0.068–0.210)	**<0.001**
**6-month mortality**		
CTP + sCD163 vs. CTP	0.144 (0.095–0.192)	**<0.001**
MELD + sCD163 vs. MELD	0.118 (0.075–0.161)	**<0.001**
ALBI + sCD163 vs. ALBI	0.138 (0.075–0.202)	**<0.001**

*P value < 0.05 was considered significant and was indicated in bold*.

*CTP, Child-Turcotte-Pugh; MELD, model for end-stage liver disease; ALBI, Albumin-Bilirubin; sCD163,soluble CD163*.

## Discussion

Liver cirrhosis is the end stage of various chronic liver diseases (18) and is mainly caused by HBV infection in China (19). This is in accordance with the results of our study, in which the most common etiology of liver cirrhosis was HBV infection (63.5%). A previous study showed that 3–5% of compensated cirrhosis patients develop DeCi each year, and the 5-year survival rate of DeCi is approximately 14–35% (20). Consistent with a previous study on patients with DeCi, in the present study, approximately 16.0% of the patients with DeCi died within 28 days, 24% died within 3 months, and 28% died within 6 months (21–23). DeCi imposes a substantial economic burden on families and society due to repeated hospitalizations (24). Liver transplantation is an effective treatment for DeCi, but the large number of patients requiring transplantation and the scarcity of available donor livers have limited its clinical application (25). Therefore, it is important to assess the prognosis of DeCi patients in the early stage. Currently, many prognostic models are used to predict the outcome in DeCi patients, such as the CTP score, MELD score and ALBI score. The CTP score has been utilized as an effective tool for the assessment of the prognosis of cirrhosis for decades (26). However, its inherent deficiencies reduce its reliability. In addition, the CTP score includes HE and ascites, which are not consistently evaluated during clinical examinations. The MELD score incorporates only three objective variables (total bilirubin, creatinine and the INR), and it is susceptible to confounding by the presence of hemorrhage and ascites and the use of diuretics (27). The ALBI score includes only two factors, and it has been reported to be useful for the prediction of outcomes in patients with primary biliary cirrhosis (28). The ALBI, MELD and CTP scores have similar efficacy in terms of predicting mortality in DeCi patients (29). However, these prognostic models are based on parameters that do not include markers of disease pathogenesis or progression. Therefore, we investigated pathogenic biomarkers in DeCi patients and further improved the original scoring systems.

CD163 is mainly expressed on the surface of macrophages. It is a hemoglobin-haptoglobin scavenger receptor and is released into circulation as soluble (s)CD163 after it is shed from immune cells (30). sCD163 has been considered a marker of macrophage activation, and the level of sCD163 correlates with the severity of liver disease (31, 32). The results of our study were consistent with previous results: the level of sCD163 was significantly higher in non-surviving patients than in surviving patients. Furthermore, we found positive correlations between the sCD163 level and the CTP, MELD and ALBI scores, which reflect the severity of liver cirrhosis. Similarly, a previous study confirmed that the sCD163 level increased with increased CTP scores in patients with cirrhosis (13, 33). With increasing severity of liver disease, the activation of macrophages also increases. When macrophages are activated by inflammation or infection, systemic inflammation and even ACLF can develop as a result of exaggerated immune responses and cytokine storms (34). Macrophage activation by pathogen-associated molecular patterns (PAMPs) and damage-associated molecular patterns (DAMPs) is involved in immunological processes during inflammation (35). PAMPs and DAMPs can collectively contribute to the development of systemic inflammation in patients with a variety of liver diseases (36). Interferon-γ (IFN-γ) and the cytokine interleukin-12 (IL-12) are known to initiate a proinflammatory response in macrophages (37). Furthermore, IFN-γ3 is recognized as a promoter of hepatic inflammation and the progression of fibrosis (38). Hepatic inflammation and the progression of liver fibrosis are associated with a poor prognosis in cirrhosis patients. ACLF occurs in patients with pre-existing liver disease, is thought to be associated with systemic inflammation and is associated with a high mortality rate (39). Younossi et al., and Ekstedt et al., showed that the presence of advanced fibrosis (stage 3–4) was associated with increased risks of overall and liver disease-related mortality (40, 41). This highlights the important role of hepatic macrophage activation in the pathophysiology and progression of liver disease.

The main finding is the improvement in the predictive ability of the original scoring systems for mortality in DeCi patients after the inclusion of the sCD163 level. The results of the present study indicate that the sCD163 level had the highest prognostic value for mortality in DeCi patients at 28 days, 3 months and 6 months, and when sCD163 was added to the original scores, their predictive value significantly improved. In a multivariate logistic regression analysis of the predictors of survival, sCD163 was identified as an independent predictor. sCD163 reflects systemic monocyte/macrophage activation. Monocyte/macrophage activation was found to be elevated in patients with pneumonia and sepsis, and the highest levels were found in patients with liver disease (42, 43). Elevated plasma concentrations of sCD163 reflect the activation of hepatic macrophages (12), and they may therefore correlate well with disease severity and prognosis. A prospective study evaluated the predictive value of sCD163 and found that an elevated sCD163 level was an independent risk factor for variceal bleeding and long-term mortality in patients with cirrhosis (9). Zhao R et al., designed a prospective cohort study and found that the plasma sCD163 level was an independent prognostic factor for short-term mortality in patients with HBV-ACLF, and its inclusion improved the predictive value of the clinical scoring models (44). Recent studies have reported the prognostic value of sCD163 for the prediction of long-term survival in DeCi patients (45). Our study indicates that sCD163 may be valuable as a disease marker for the prediction of short-term and long-term outcomes in DeCi patients, which highlights a key role of macrophage activation in the development and progression of DeCi.

Our study has some limitations. First and most importantly, our study was a single-center study. Second, the results in our study were not validated. Third, dynamic changes in sCD163 levels during hospitalization in patients with DeCi were not examined. Thus, further studies are needed to investigate the influence of dynamic changes in sCD163 levels among patients with DeCi.

In conclusion, we demonstrated that the inclusion of sCD163, a specific marker of macrophage activation, improved the ability of the original scores to predict the outcome in DeCi patients.

## Data Availability Statement

The original contributions presented in the study are included in the article/Supplementary Material, further inquiries can be directed to the corresponding author/s.

## Ethics Statement

The studies involving human participants were reviewed and approved by Study protocol was approved by the ethics committee of First Affiliated Hospital of Nanchang University (No. IIT[2017]009). The patients/participants provided their written informed consent to participate in this study. Written informed consent was obtained from the individual(s) for the publication of any potentially identifiable images or data included in this article.

## Author Contributions

YZ: designed and writing—original draft. CH and YN: writing—original draft. QL: data analysis. NX: prepared figures and tables. LL: data collection. XZ: conceptualization, funding acquisition, supervision, and writing—review and editing. All authors contributed to the article and approved the submitted version.

## Conflict of Interest

The authors declare that the research was conducted in the absence of any commercial or financial relationships that could be construed as a potential conflict of interest.
