# Supplementary material for: Soluble CD163 Is a Predictor of Mortality in Patients With Decompensated Cirrhosis
Source: Front Med (Lausanne). 2021 Jul 15;8:698502. doi: 10.3389/fmed.2021.698502 (PMC8319469; doi:10.3389/fmed.2021.698502)
Supplement: Supplementary file 1 [file Table_1.DOCX]

| Cause of death | 6-months (n=99) |
| --- | --- |
| Hemorrhagic shock, n (%) | 45(45.5%) |
| Respiratory failure, n (%) | 15(15.2%) |
| Pulmonary infection, n (%) | 10(10.1%) |
| Hepatic encephalopathy, n (%) | 17(17.2%) |
| Ruptured thoracic aortic aneurysmn , n (%) | 1(1.0%) |
| Acute myocardial infarction, n (%) | 1(1.0%) |
| cardiogenic shock, n (%) | 7(7.0%) |
| Uncertain, n (%) | 3(3.0%) |

Supplement table 1: Causes of death in Deci patients

**Abbreviations**: Deci: decompensated cirrhosis.
